# Genetic Mapping of a new *Hippo* allele, *Hpo^N.1.2^,* in *Drosophila melanogaster*

**DOI:** 10.17912/micropub.biology.000383

**Published:** 2021-04-08

**Authors:** Jamie L Siders, Kayla L Bieser, Danielle R Hamill, Erika C Acosta, Olivia K Alexander, Humza I Ali, Micah J Anderson, Hayden R Arrasmith, Mustafa Azam, Nikki J Beeman, Hassan Beydoun, Lauren J Bishop, Morgan D Blair, Brianna Bletch, Heather R Bline, Jennifer C Brown, Kelly M Burns, Karina C Calagua, Lexie Chafin, William AH Christy, Carlyn Ciamacco, Hannah Cizauskas, Caitlyn M Colwell, Abigail R Courtright, Lucero Diaz Alavez, Rayne IS Ecret, Fatima Edriss, Taylor G Ellerbrock, Madison M Ellis, Erica M Extine, Eric Feldman, Luke J Fickenworth, Caroline M Goeller, Alexis S Grogg, Yailine Hernandez, Abigail Hershner, Megan M Jauss, Leyre Jimenez Garcia, Katey E Franks, Ethan T Kazubski, Emily R Landis, Jon Langub, Tia N Lassek, Triet C Le, Julia M Lee, Daniel P Levine, Phoebe J Lightfoot, Natasha Love, Ali Maalhagh-Fard, Colin Maguire, Brynna E McGinnis, Bhargavi V Mehta, Veronica Melendrez, Zimri E Mena, Seth Mendell, Petra Montiel-Garcia, Autumn S Murry, Riley A Newland, Ryan M Nobles, Neha Patel, Yashodhara Patil, Cassidy L Pfister, Victoria Ramage, Mya R Ray, Joseph Rodrigues, Victoria C Rodriquez, Yara Romero, Alexandra M Scott, Nicholas Shaba, Samantha Sieg, Kayla Silva, Sahiba Singh, Aleksandria J Spargo, Savanna J Spitnale, Nicole Sweeden, Logan Tague, Breanna M Tavernini, Kathleen Tran, Liselle Tungol, Kylie A Vestal, Amber Wetherbee, Kayla M Wright, Anthony T Yeager, Rehab Zahid, Jacob D Kagey

**Affiliations:** 1 School of Science, Technology, and Mathematics, Ohio Northern University, Ada, OH USA; 2 Department of Physical and Life Sciences, Nevada State College, Henderson, NV USA; 3 Department of Zoology, Ohio Wesleyan University, Delaware, OH USA; 4 Biology Department, University of Detroit Mercy, Detroit, MI USA; 5 ReBUILDetroit, University of Detroit Mercy, Detroit, MI USA

## Abstract

Genetic screens provide a mechanism to identify genes involved with different cellular and organismal processes. Using a Flp/FRT screen in the *Drosophila *eye we identified mutations that result in alterations and de-regulation of cell growth and division. From this screen a group of undergraduate researchers part of the Fly-CURE consortium mapped and characterized a new allele of the gene *Hippo*, *Hpo^N.1.2^.*

**Figure 1. The Dark82, HpoN.1.2 mosaic eye results in dramatic tissue overgrowth and pupal lethality.**
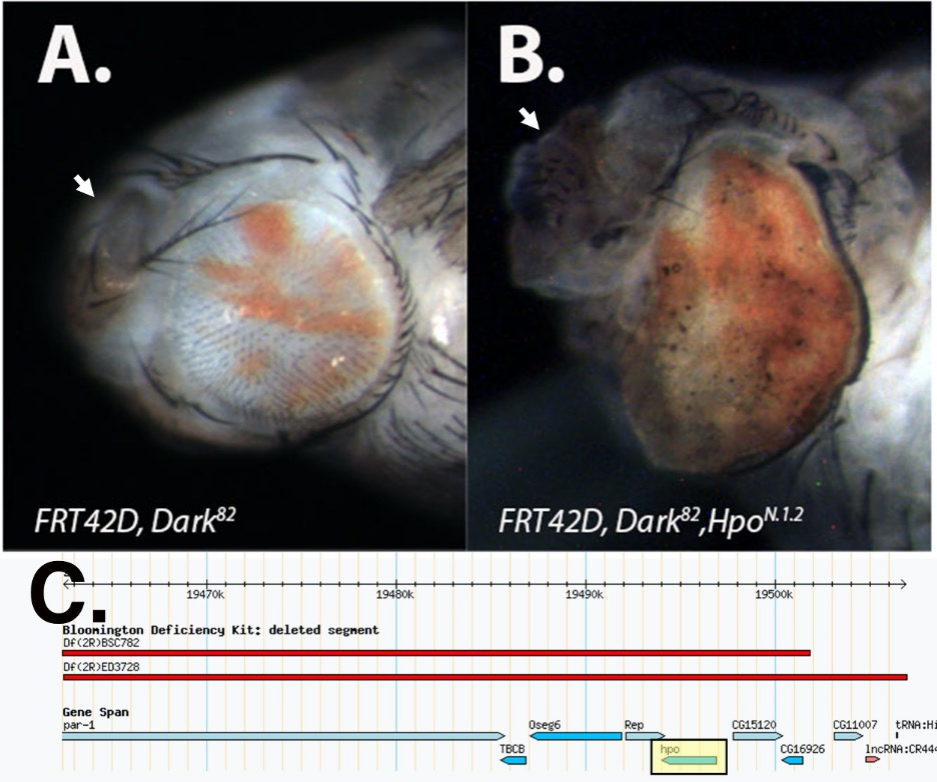
A.) Lateral view of *FRT42D, Dark^82^* mosaic control eye and B.) *FRT42D, Dark^82^,Hpo^N.1.^*^2 ^mosaic mutant pupal eyes at the same magnification (40x). In both genotypes, mutant tissue displays red pigmentation (w^+mC^). Arrows on A and B point to antennae, eyes are oriented with anterior to the left and dorsal to the top. C.) Genomic region of chromosome 2R in which mutant *N.1.2* failed to complement (2R:19,462,450..19,506,861). The *N.1.2* mutation mapped to the *hpo* gene locus found in this region (yellow highlight). Image adapted from flybase.org (Gramates 2017).

## Description

In order to identify conditional regulators of cell growth and tumorigenesis, an EMS-based genetic screen was conducted in *Drosophila* *melanogaster* utilizing the Flp/FRT system in an apoptotic null background (Akdemir *et al.*, 2006). A fly line possessing the *FRT42D, Dark^82^* chromosome and containing a mini-white cassette (w^+mC^) was used for EMS mutagenesis. Subsequent matings to *FRT42D; Ey-Flp* flies facilitated phenotypic screening in eye tissue. Phenotypes observed in the screen included alterations in mosaicism (red > white pigmentation pattern), eye and/or antennal overgrowth, defects in patterning, and pupal lethality (Kagey *et al.* 2012). One of the pupal lethal mutants identified in this screen, *N.1.2,* is discussed here. Genetic crosses between *FRT42D, Dark^82^, N.1.2* X *FRT42D; Ey-Flp* resulted in near complete pupal lethality (~ 90%) due to dramatic tissue overgrowth of the eye, antennae, and inter-ocular space (Figure 1B compared to 1A). Due to the pupal lethality associated with the eye tissue overgrowth, flies were dissected from late pupal stages in order to visualize the phenotype. Imaging following dissection shows that the mutant eyes are comprised of mostly mutant (pigmented) tissue that protrudes from the eye cavity creating tissue folding (Figure 1B). Control *FRT42D, Dark^82^* flies exhibited a balance in the red:white ratio and no eye overgrowth (Figure 1A). For direct comparison to *N.1.2*, the *FRT42D, Dark^82^* mosaic eye was also imaged at the late pupal stage. Eyes were imaged under 70% ethanol on an AM Scope digital camera at 40x using a LED light ring.

In order to map the genetic location of the N.1.2 mutation, we conducted complementation tests with deficiency strains, and looked for lethality, consistent with the phenotype of homozygous mutant flies of the N.1.2 stock. Deficiency crosses were conducted by mating *FRT42D, Dark^82^, N.1.2/CyO* virgin females to males from each of 86 deficiency stocks with deletions distal to the *FRT42D* site on the right arm of chromosome two. All stocks used for mapping were part of the Bloomington Deficiency 2R Kit (Cook et. al., 2012). The genetic mapping was conducted by undergraduate research students from Nevada State College, Ohio Northern University, Ohio Wesleyan University, and the University of Detroit Mercy as part of the Fly-CURE consortium (Vrailas-Mortimer *et al.* 2021; Bieser *et al.* 2019; Stamm e*t al.* 2019). The *N.1.2* mutant failed to complement two of these deficiencies, *Df(2R)BSC782* (2R: 19,451,027..19,501,804) and *Df(2R)ED3728* (2R:19,462,450..19,726,747). These two deficiencies overlapped from cytological bands 56D10-56D14 (2R:19,462,450..19,506,861) and included the hippo (*hpo*) gene locus and nine other protein coding genes (2R:19,492,996..19,496,856) (Figure 1C). Subsequent crosses to individual lethal alleles of *hpo*: *hpo^MGH1^*and *hpo^KS240^*, failed to complement *N.1.2*, confirming that the *N.1.2* mutant is a newly isolated *hpo* allele, *hpo^N.1.2^* (Harvey *et al.*, 2003; Udan *et al.*, 2003).

The *hpo* gene functions as a negative regulator of cell growth and is a part of a highly conserved signaling pathway first characterized in *Drosophila* (reviewed in Harvey and Hariharan 2012). Overall, research has shown that the hippo pathway is critical for regulating organ size through regulation of apoptosis, cell survival, cell polarity, and cell proliferation (Harvey et.al., 2003; Jia et.al. 2003; Pantalacci et. al., 2003; Udan et. al., 2003; reviewed in Yu et. al, 2015). Consistent with the eye phenotypes observed in the *N.1.2* mutation, mutations in *hpo* are known to result in striking overgrowth phenotypes in a variety of tissue types from flies to humans (Pan 2010, Pluoffe *et al.*, 2015; Yu et. al., 2015). Identification of this novel *hpo^N.1.2^* allele will support further research into the molecular mechanisms by which multicellularity is regulated and restricted by this critical signaling pathway.

## Reagents

*FRT42D, Dark^82^/CyO* (Akdemir *et al.*, 2006)

*FRT42D, Dark^82^, hpo^N.1.2^/CyO* (this manuscript)

*FRT42D; Ey-Flp* (BDSC 8211)

Bloomington Drosophila Stock Center 2R Deficiency Kit(Cook **et al.*,* 2012)

*w^1118^; Df(2R)BSC782/SM6a* (BDSC 27354)

*w^1118^; Df(2R)ED3728/SM6a* (BDSC 9067)

*yw; FRT42D, hpo^KS240^/CyO* (BDSC 25085)

*hpo^MGH1^* (Harvey *et al.* 2003)
